# Rbm15-Mkl1 Interacts with the Setd1b Histone H3-Lys4 Methyltransferase via a SPOC Domain That Is Required for Cytokine-Independent Proliferation

**DOI:** 10.1371/journal.pone.0042965

**Published:** 2012-08-21

**Authors:** Jeong-Heon Lee, David G. Skalnik

**Affiliations:** 1 Wells Center for Pediatric Research, Departments of Pediatrics and Biochemistry and Molecular Biology, Indiana University School of Medicine, Indianapolis, Indiana, United States of America; 2 Department of Biology, Indiana University-Purdue University Indianapolis School of Science, Indianapolis, Indiana, United States of America; INSERM UMR S_910, France

## Abstract

The Rbm15-Mkl1 fusion protein is associated with acute megakaryoblastic leukemia (AMKL), although little is known regarding the molecular mechanism(s) whereby this fusion protein contributes to leukemogenesis. Here, we show that both Rbm15 and the leukemogenic Rbm15-Mkl1 fusion protein interact with the Setd1b histone H3-Lys4 methyltransferase (also known as KMT2G). This interaction is direct and requires the Rbm15 SPOC domain and the Setd1b LSD motif. Over-expression of Rbm15-Mkl1 in the 6133 megakaryoblastic leukemia cell line, previously established by expression of the Rbm15-Mkl1 fusion protein in mice (Mercher et al., [2009] J. Clin. Invest. 119, 852–864), leads to decreased levels of endogenous Rbm15 and increased levels of endogenous Mkl1. These cells exhibit enhanced proliferation and cytokine-independent cell growth, which requires an intact Rbm15 SPOC domain that mediates interaction between the Rbm15-Mkl1 fusion protein and the Setd1b methyltransferase. These results reveal altered Setd1b complex function and consequent altered epigenetic regulation as a possible molecular mechanism that mediates the leukemogenic activity of the Rbm15-Mkl1 fusion protein in AMKL.

## Introduction

Acute megakaryoblastic leukemia (AMKL) is a subtype of acute myeloid leukemia with a poor prognosis. AMKL is frequently found in children with Down syndrome, where it is associated with mutation of the GATA1 transcription factor [1]. A second form of AMKL is associated with the reciprocal t(1;22)(p13;q13) chromosomal translocation in infants and young children [2], [3]. This rearrangement generates a transcript encoding all of the putative functional domains of both RNA binding motif 15 (Rbm15) (also known as Ott) and megakaryoblastic leukemia (Mkl) 1 (also known as Mal, Bsac, and Mrtf-a) [4], [5]. Rbm15 is a member of the split ends (Spen) family of proteins, which are characterized by three N-terminal RNA recognition motifs (RRM) and a Spen paralogs and orthologs C-terminal (SPOC) domain [6]. The Spen family of proteins plays a role in cell fate specification during development by regulating transcription of developmental control genes [7]. For example, SHARP (SMRT/Hdac1-associated repressor protein), the human homolog of the *Drosophila* Spen protein, is a component of transcriptional repression complexes in both nuclear receptor and Notch/Rbp-jk signaling pathways [8], [9]. Rbm15 is a regulator of both transcription and mRNA export, and is required for normal embryonic development and hematopoietic stem cell function [10]–[16]. The other fusion partner, Mkl1, is a member of the myocardin family of transcriptional co-activators. Mkl1, as well as the Rbm15-Mkl1 fusion protein, activate a subset of endogenous serum response factor (SRF) target genes in an SRF-dependent manner in non-hematopoietic cell lines [17]–[19].

Yeast Set1 is the enzymatic component of a multimeric histone methyltransferase (HMT) complex that catalyzes the methylation of histone H3-Lys4, an epigenetic modification generally associated with transcriptional activity [20]. Although yeast cells express only a single Set1 protein, mammalian cells encode numerous Set1-like enzymes, including Setd1a, Setd1b, mixed-lineage leukemia (Mll) 1, Mll2, and Mll3/4 [21]. Many of these enzymes have been implicated as critical epigenetic regulators of development. For example, rearrangements of the *MLL1* gene are associated with aggressive acute leukemia in both children and adults [22]; the *MLL2* gene is amplified in some solid tumors [23]; and the *MLL3* gene is deleted in cases of myeloid leukemia [24]. Menin, a targeting component of the Mll1 and Mll2 complexes, is mutated in heritable multiple neoplasia type I [25], and Ash2 is a core component of the Set1-like HMT complexes that shows elevated expression in human tumors and tumor cell lines [26]. Molecular analysis of *MLL1*-rearranged leukemia indicates that leukemogenic Mll1-fusion proteins create epigenetic signatures that persistently activate developmental regulator genes. These genes escape from the silencing that occurs during normal hematopoietic differentiation, leading to perturbed cellular specification and cellular transformation [22], [27]–[29].

Importantly, a knock-in allele of Rbm15-Mkl1 causes abnormal fetal megakaryopoiesis in mice and causes AMKL in adult animals at a low frequency [30]. However, the molecular mechanisms whereby the oncogenic Rbm15-Mkl1 fusion protein induces AMKL are largely unknown. We report that Rbm15-Mkl1 specifically associates with the Setd1b HMT complex via its SPOC domain, which is required for Rbm15-Mkl1-mediated aberrant cellular proliferation and transformation.

## Materials and Methods

### Cloning of Full Length Human Setd1b cDNA

A partial cDNA encoding the C-terminal half (aa 1120–1923) of human Setd1b was previously described [31]. The cDNAs encoding the N-terminal and middle regions of human Setd1b were synthesized by RT-PCR from human embryonic kidney (HEK) 293 cells and cloned into pBluescript (Stratagene). The full length cDNA of human Setd1b was assembled by combining partial Setd1b cDNAs using restriction enzyme digestion and ligation. The full-length cDNA contains sequence that encodes an additional 43 aa at residue 1042 compared to the predicted sequence (XP_037523 and P_946855) of Setd1b, which presumably reflects an alternative splicing event. The functional significance of this difference is not known. The new cDNA sequence of full length Setd1b has been deposited in Genbank (accession # JF813787).

### Plasmid Construction

Setd1a expression constructs were previously described [32]. The murine Rbm15 cDNA (pcDNA3-NT-GFP-OTT) was kindly provided by Dr. Diane Krause (Yale University). Expression constructs for human Rbm15 (pcDNA3 5′FLAG-Rbm15) and human Rbm15-Mkl1 (pcDNA3 Rbm15-Mkl1-3′FLAG) were kindly provided by Dr. Ron Prywes (Columbia University). The cDNAs encoding human Setd1b, Rbm15, Rbm15-MKL1, and Mkl1 were subcloned into pcDNA3 or pcDNA5/TO vectors (Invitrogen) carrying an N-terminal FLAG or Myc epitope using PCR and restriction enzyme digestions. Site-directed mutagenesis was performed on the SPOC domain of Rbm15 and the LSD motif of Setd1b using the QuickChange site-directed mutagenesis kit (Stratagene) in accordance with the protocol provided by the manufacturer. The nucleotide sequence of all constructs was confirmed by DNA sequencing.

### Cell Lines

HEK293 cells were cultured and transfected as described [33]. A T-REx HEK293 cell line (Invitrogen) that constitutively expresses the tetracycline repressor was maintained, transfected, and selected as previously described [31]. Established inducible T-REx HEK293 cell lines carrying each expression construct were maintained in Dulbecco’s modified Eagle’s medium supplemented with 10% bovine calf serum, 50 ug/ml hygromycin B, and 5 ug/ml blasticidin. The murine 6133 megakaryoblastic leukemic cell line [30] was kindly provided by Dr. Thomas Mercher (Université René Descartes, Paris, France) and was cultured in RPMI 1640 medium containing 10% FBS, 100 U/ml of penicillin-streptomycin, and 10 ng/ml SCF as described [30].

### Mass Spectrometry

T-REx HEK293 cells that inducibly express FLAG-Setd1a (aa 247–1408), FLAG-Setd1b (aa 225–1671), or empty vector were treated with 0.5 ug/ml doxycycline for 3 days. Nuclear extracts were prepared in extraction buffer (10 mM PIPES pH 7.2, 300 mM sucrose, 3 mM MgCl_2_, 1 mM EGTA, 0.5% Triton X-100) supplemented with protease inhibitors as previously described [34]. Anti-FLAG M2 agarose beads (Sigma) were added and incubated for 3 h. Bound proteins were eluted by 250 ug/ml FLAG peptide after extensive washing. Proteins were separated by SDS-PAGE after denaturation and stained by Coomassie Brilliant Blue. Protein bands were excised and processed for protein identification by an LTQ ion trap mass spectrometer in the Protein Analysis Research Center (IU School of Medicine). Protein identity was determined by searching tandem mass spectra against the IPI human protein database with SEQUEST and X!Tandem as previously described [35].

### Immunoprecipitation and Western Blotting

Preparation of nuclear or whole cell extracts, immunoprecipitation, and Western blotting analysis were performed as previously described [31], [32]. Where indicated, samples were treated with 50 ug/ml of RNase A for 15 min at 37°C prior to analysis, as previously described (11,12). Antisera utilized for immunoprecipitation and Western blotting are as follows: anti-FLAG and anti-actin antibodies were obtained from Sigma; anti-Rbm15 antibodies were obtained from Bethyl Laboratories or Proteintech Ltd.; anti-Ash2, anti-Rbbp5, and anti-Setd1b antibodies were obtained from Bethyl Laboratories; anti-H3K4me3 and anti-histone H3 antibodies were obtained from Abcam; anti-H3K9me2 were obtained from Upstate Biotechnology; anti-Myc antibody was obtained from Santa Cruz Biotechnology; and anti-Mkl1 antiserum was kindly provided by Dr. Paul Herring (Indiana University). Generation of antisera directed against Setd1a, Setd1b, Cfp1, Wdr5, and Wdr82 was previously described [31].

### Histone Methyltransferase Assay

Histone methyltransferase assays were performed as previously described [31], [34]. To assess methyltransferase specificity, reaction products containing recombinant histone H3 substrate were analyzed by Western blotting using modification-specific antibodies.

### In vitro GST Pull-down Assay

The cDNA encoding the SPOC domain of human Rbm15 (782–977 aa) was subcloned into the pBAD/His vector (Invitrogen) and was subsequently transformed into E. coli. Recombinant protein was induced by 0.1% L-arabinose for 4 h at room temperature and was purified using Ni-NTA agarose (Qiagen) according to the manufacturer’s instructions. Purified recombinant protein was dialyzed against PBS. The cDNA encoding the LSD domain of human Setd1b (542–628 aa) was subcloned into the pGEX 4T vector (Pharmacia Ltd.), and GST or GST-LSD proteins were purified as previously described (32). For analysis of protein-protein interactions *in vitro*, purified proteins were incubated in 1 ml of pull-down buffer (20 mM Tris-HCl pH 8.0, 150 mM NaCl, 2 mM EDTA, 0.2% NP-40, and 0.5% deoxycholate) for 2 h, and glutathione-agarose beads (Sigma) were added and incubated for 1 h. Beads were extensively washed with pull-down buffer and bound proteins were denatured by SDS sample buffer. Proteins were separated by SDS-PAGE and analyzed by Western blotting or Coomassie staining.

### Viral Production, Transduction, and Cell Sorting

The cDNAs encoding Rbm15, Rbm15 (K795A), Rbm15-Mkl1, and Rbm15-Mkl1 (K795A) were subcloned into the lentiviral pCL6IEGwo expression vector. The pCL6IEGwo vector was kindly provided by Dr. Helmut Hanenberg (Indiana University) and possesses a 5′ CMV promoter, an internal ribosomal entry site (IRES)-driven enhanced green fluorescence protein (GFP) coding sequence, and 3′ Woodchuck element. The pCL6IEGwo vectors carrying each expression construct were transiently transfected into HEK293T cells along with helper plasmids pCD/NL-BH and pMDG.1 by calcium phosphate co-precipitation. Media was changed 6 h after transfection and the viral supernatant was harvested at 24 and 48 h post-transfection. Viral particles were concentrated 10-fold by centrifugation for 3 h at 12,000 rpm with a Beckman SW28 rotor (Beckman Coulter, Fullerton, CA). Resultant virus was aliquoted and stored at -80°C until use. Murine 6133 megakaryoblastic leukemic cells were transduced by incubation with viral supernatants overnight. Cells were cultured with fresh media for 72 hours before sorting GFP-positive cells on a FACSAria cell sorter in the Flow Cytometric Resource Facility (IU School of Medicine). GFP-positive cells were cultured in the presence of SCF for several days, and then 40,000 cells were plated in the presence or absence of SCF and cell proliferation was analyzed by enumeration following staining with trypan blue.

### Flow Cytometric Analysis of Apoptosis

For apoptosis analysis, cells were harvested, washed once with cold PBS, and resuspended in 100 ul binding buffer (10 mM HEPES pH 7.4, 140 mM NaCl, and 2.5 mM CaCl_2_). Annexin V–APC (Pharmingen, San Diego, CA) and 7-AAD were added and incubated at room temperature for 15 min in the dark. Binding buffer (400 ul) was then added prior to flow cytometric analysis using a FACS Calibur flow cytometer (Becton Dickinson, San Jose, CA). Apoptotic cells were defined as the fraction of Annexin V–positive and 7-AAD-negative cells.

## Results

### The Setd1b HMT Complex Associates with Rbm15

**Figure 1:**
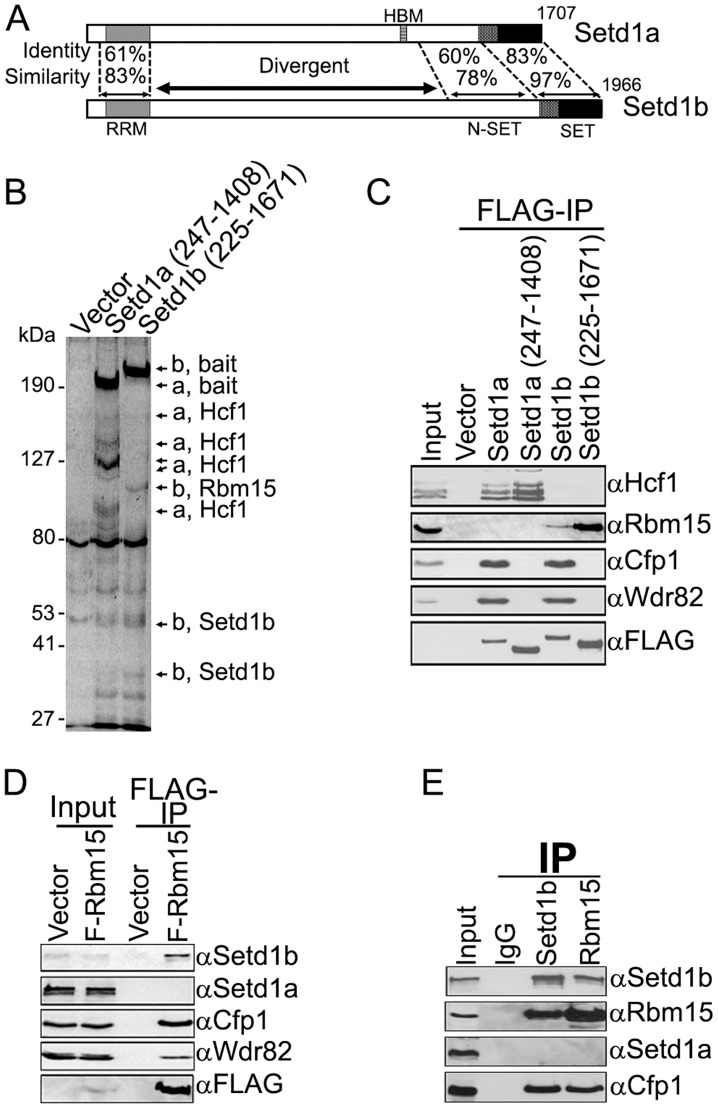
Rbm15 interacts with the Setd1b HMT complex. A. The homology between Setd1a and Setd1b is shown. Areas of identity and similarity between human Setd1a (NP_055527) and Setd1b (JF813787) were determined by ClustalW (1.82) (43). RRM, RNA recognition motif; HBM, Hcf1 binding motif; N-SET, N-terminal region of the SET domain; SET, catalytic SET histone methyltransferase domain. Numbers indicate amino acid residues of human Setd1a and Setd1b proteins. B. Nuclear extracts were isolated from inducible T-REx HEK293 cell lines that express FLAG-tagged versions of the central divergent domains of Setd1a or Setd1b (or carrying the empty expression vector). Extracts were subjected to FLAG immunoprecipitation and bound proteins were eluted by FLAG peptide after extensive washing. Proteins were analyzed by SDS-PAGE and stained with Coomassie Blue. Arrows indicate proteins identified by mass spectrometry, and “a” or “b” in front of each identified protein indicates Setd1a and Setd1b interactions, respectively. C. Constructs that express FLAG-tagged full length Setd1a, Setd1a middle fragment, full length Setd1b, or Setd1b middle fragment (or empty vector) were transiently transfected into HEK293 cells. Nuclear extracts were isolated and subjected to FLAG immunoprecipitation, and immunoprecipitates were analyzed by Western blotting using the indicated antisera. D. HEK293 cells were transiently transfected with a FLAG-Rbm15 vector or empty expression vector. Nuclear extracts were isolated and subjected to FLAG immunoprecipitation, and immunoprecipitates were analyzed by Western blotting using the indicated antisera. E. Nuclear extracts isolated from HEK293 cells were subjected to immunoprecipitation (IP) using antisera directed against Setd1b or Rbm15. Immunoprecipitates were analyzed by Western blotting using the indicated antisera.

We previously identified two human Set1-like HMT complexes [31], [34]. These multimeric complexes are identical except for the enzymatic methyltransferase subunit (Setd1a or Setd1b). Importantly, confocal microscopy reveals that Setd1a and Setd1b exhibit a largely non-overlapping sub-nuclear distribution, strongly suggesting distinct functions for these closely related histone H3-Lys4 methyltransferases [31]. The N-terminal and C-terminal regions of Setd1a and Setd1b are highly homologous, and serve as docking sites for common components of the multimeric HMT complexes [31], [32] (Fig. 1A). However, the middle portions of the Setd1 proteins are divergent [31], suggesting that these regions may play a role in differential genomic targeting of Setd1 HMT complexes via interaction with distinct targeting proteins. To investigate this further, FLAG immunoprecipitation was performed using nuclear extracts isolated from HEK293 cell lines that stably express FLAG-tagged middle fragments of Setd1a or Setd1b, and recovered proteins were identified by mass spectrometry. As expected, host cell factor 1 (Hcf1) co-purifies with the middle fragment of Setd1a, presumably via interaction with the Hcf1 binding motif (HBM), as previously reported [32], [36] (Fig. 1B). Importantly, a ∼100 kDa protein co-immunoprecipitates specifically with the Setd1b middle fragment, and this protein was identified by mass spectrometry as Rbm15 (Fig. 1B).

To further evaluate the authenticity and specificity of identified interacting proteins, constructs that express FLAG-tagged full-length Setd1a, Setd1a middle fragment, full-length Setd1b, or Setd1b middle fragment were transiently expressed in HEK293 cells and protein interactions were analyzed by FLAG immunoprecipitation. Figure 1C demonstrates that Setd1a specifically interacts with endogenous Hcf1, and Setd1b specifically interacts with endogenous Rbm15. The middle fragments of Setd1a and Setd1b interact with Hcf1 or Rbm15, respectively, but not with common components (Cfp1 and Wdr82) of the Setd1 HMT complexes, indicating that the interactions of Rbm15 with Setd1b and Hcf1 with Setd1a are not dependent on assembly of intact Setd1 HMT complexes. Furthermore, FLAG-Rbm15 specifically interacts with endogenous Setd1b but not with Setd1a (Fig. 1D). Rbm15 also co-immunoprecipitates the Cfp1 and Wdr82 components of the Setd1 HMT complexes, indicating that Rbm15 associates with the intact Setd1b HMT complex. To investigate the interaction between endogenous Rbm15 and Setd1b proteins, nuclear extracts isolated from HEK293 cells were immunoprecipitated using Rbm15 and Setd1b-specific antibodies. Figure 1E demonstrates that endogenous Setd1b and Rbm15 immunoprecipitate each other and Cfp1, but not Setd1a in HEK293 cells. These results indicate that Rbm15 specifically interacts with the divergent middle region of Setd1b.

### The SPOC Domain of Rbm15 is Required for Interaction with Setd1b

**Figure 2:**
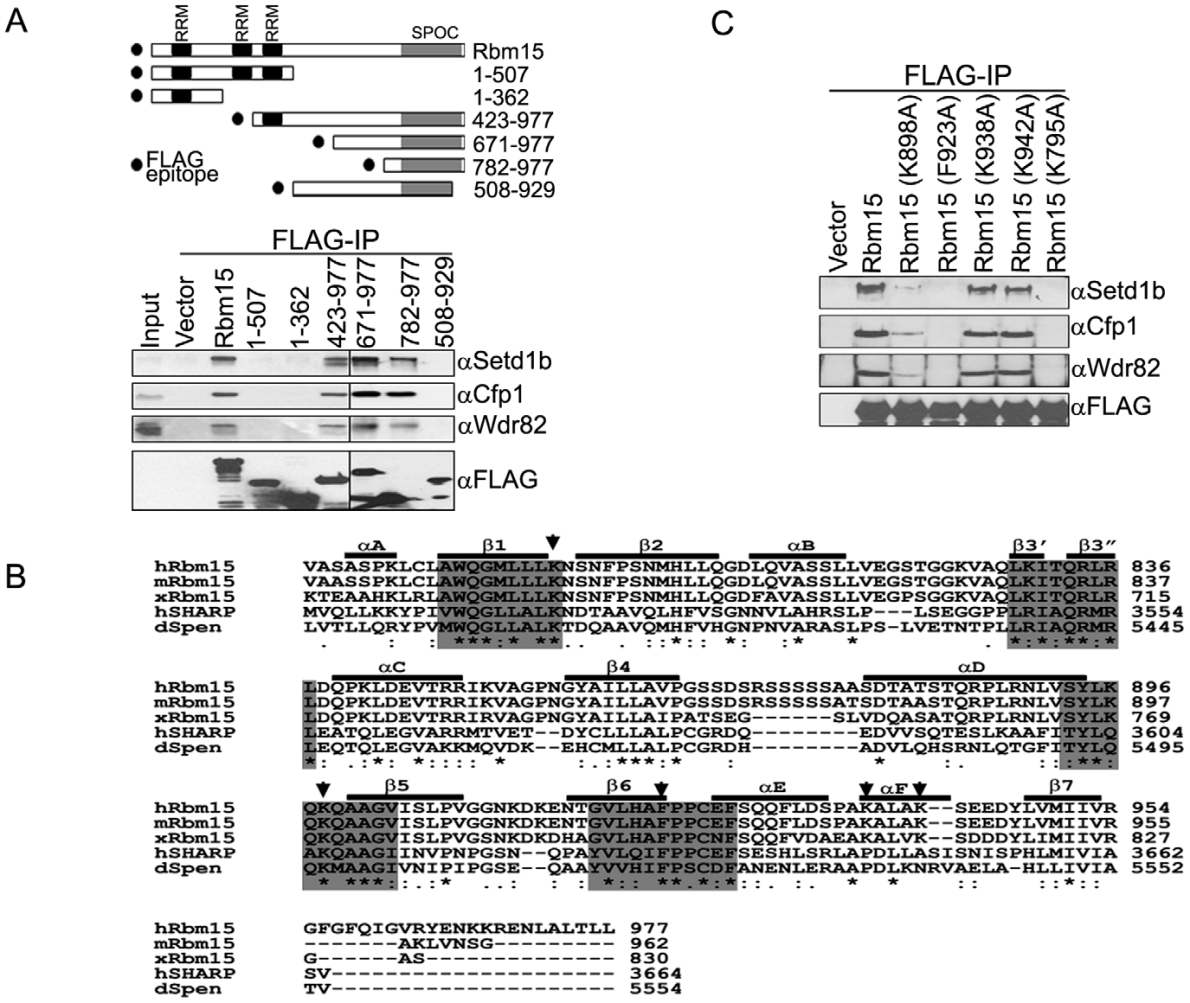
The SPOC domain of Rbm15 interacts with the Setd1b complex. A. Schematic diagram of various Rbm15 expression constructs are shown. Numbers indicate amino acid residues. HEK293 cells were transiently transfected with various expression constructs of Rbm15 fragments carrying an N-terminal FLAG epitope. Whole cell extracts were prepared and subjected to FLAG immunoprecipitation, and immunoprecipitates were analyzed by Western blotting using the indicated antisera. B. SPOC domains are highly conserved in *Spen* family proteins. Primary sequences of SPOC domains from human Rbm15, mouse Rbm15, xenopus Rbm15, human SHARP, and drosophila *Spen* proteins were aligned. Secondary structures of SPOC domains were adapted from the previously published SHARP SPOC domain (37). Amino acids that form the pocket of basic patches in SPOC domains are shaded with gray boxes, and arrows indicate amino acid residues that were mutated in the experiment. C. Expression vectors encoding various point mutants of Rbm15 were transiently transfected into HEK293 and analyzed as described as above.

To further understand the basis for interaction between Rbm15 and Setd1b, FLAG immunoprecipitations were performed using various FLAG-tagged deletion fragments of Rbm15. Figure 2A shows that Rbm15 fragments that contain an intact SPOC domain, including a fragment containing as little as aa 782 to 977, immunoprecipitate endogenous Setd1b, Cfp1, and Wdr82. However, amino terminal fragments of Rbm15 that lack the entire SPOC domain, and the 508 to 929 aa fragment of Rbm15 which lacks the C-terminal region of the SPOC domain, fail to interact with Setd1b. These results indicate that the C-terminal SPOC domain of Rbm15 is necessary and sufficient for the interaction with the Setd1b HMT complex.

The SPOC domain is a highly conserved motif found within the *Spen* family of proteins [37], [38] (Fig. 2B) that interacts with repressor proteins including SMARTER, SMART, and N-CoR via interaction with LSD motifs [8], [39]. Groups of basic residues (gray shaded regions in figure 2B) fold to form a basic patch within the SPOC domain that is important for the recognition of the LSD motif [37]. To further evaluate the interaction between the Rbm15 SPOC domain and the Setd1b complex, conserved basic amino acids within the Rbm15 SPOC domain were mutated and mutants were analyzed for Setd1b interaction by FLAG immunoprecipitation and Western blotting following transfection of HEK293 cells. Figure 2C reveals that the introduction of individual mutations K898A, F923A, or K795A into the Rbm15 SPOC domain basic patch weakens or disrupts the interaction between Rbm15 and Setd1b. However, individual mutation of basic amino acids K938 or K942, which reside outside of the basic patches, do not interrupt the interaction between Rbm15 and Setd1b. These results confirm the importance of the SPOC domain for the Rbm15/Setd1b interaction and reveal mutated forms of Rbm15 that no longer interact with the Setd1b HMT complex.

### The LSD Motif of Setd1b is Required for Interaction with Rbm15

**Figure 3:**
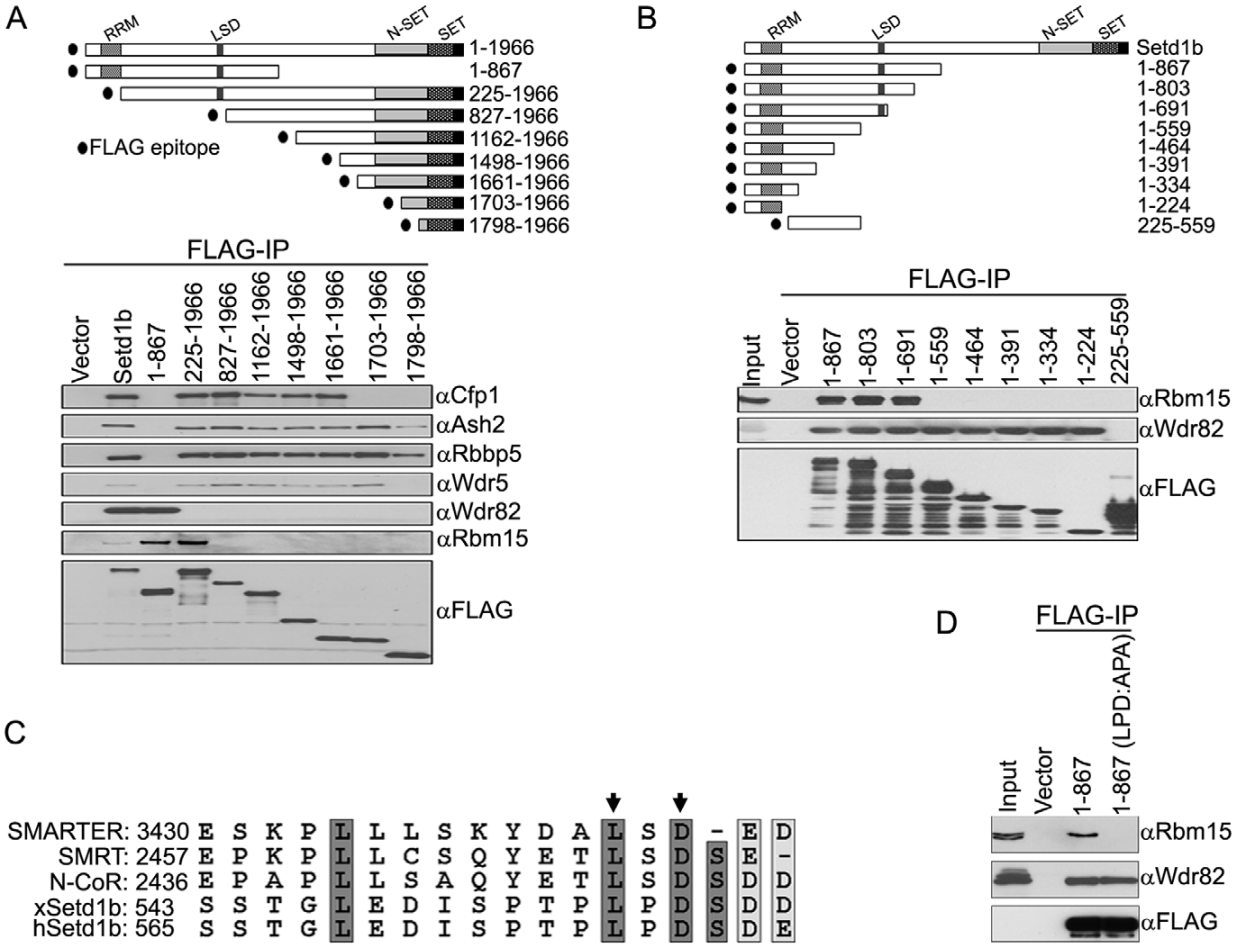
The LSD motif of Setd1b is responsible for the interaction with Rbm15. A. A schematic diagram of various Setd1b expression constructs is shown. Numbers indicate amino acid residues of the human Setd1b protein. Inducible T-REx HEK293 cell lines that express various FLAG-tagged Setd1b fragments were induced for 3 days with doxycycline. Nuclear extracts were prepared and immunoprecipitated by FLAG-IgG agarose beads, and immunoprecipitates were analyzed by Western blotting using the indicated antisera. B. HEK293 cells were transiently transfected with expression vectors encoding various FLAG-tagged deletion constructs of Setd1b. Whole cell extracts were prepared and subjected to FLAG immunoprecipitation and analyzed by Western blotting using the indicated antisera. C. Conservation of the LSD motif in SPOC domain-interacting proteins. The LSD motifs from drosophila SMARTER, human SMRT, human N-CoR, Xenopus Setd1b, and human Setd1b are aligned. Conserved amino acid residues are shaded with gray boxes. Arrows indicate the mutated amino acids in Figure 3D. D. Mutation of the LSD motif of Setd1b abolishes the interaction with Rbm15. Conserved leucine and aspartic acid residues within the Setd1B LSD motif were mutated. HEK293 cells were transiently transfected with expression vectors encoding FLAG-Setd1b fragment (1–867 aa) or FLAG-Setd1b (1–867 aa)(LPD:APA), or empty vector, and protein interactions were analyzed as described above.

To further investigate the molecular basis for interaction between Rbm15 and Setd1b, FLAG immunoprecipitations were performed following transfection of various FLAG-tagged deletion fragments of Setd1b into HEK293 cells. Figure 3A shows that the RRM domain of Setd1b interacts with the HMT complex component Wdr82, while the N-SET and SET domains of Setd1b interact with complex components Ash2, Rbbp5, Wdr5, and Cfp1, similar to what was previously shown for the Setd1a complex [32]. Rbm15 interacts with the 225 to 826 aa region of Setd1b. Further analysis of this Rbm15-interacting region using additional C-terminal deletion fragments reveals that the 560 to 691 aa region of Setd1b is required for interaction with Rbm15 (Fig. 2B). The interaction of Wdr82 with the amino terminal RRM domain of Setd1b serves as a positive control. Inspection of the Rbm15-interacting region of Setd1b reveals an evolutionarily conserved LSD motif similar to that found in repressor proteins that interact with other SPOC domain-containing proteins (Fig. 2C). Furthermore, as would be expected given the binding specificity of Rbm15 for Setd1b, the LSD motif is not found within the Setd1a protein (34). To confirm whether the LSD motif is critical for the interaction between Rbm15 and Setd1b, conserved leucine and aspartic acid residues were mutated to alanine and the mutant was analyzed by FLAG immunoprecipitation. Figure 3D demonstrates that mutation of the Setd1b LSD motif disrupts the interaction with Rbm15 without affecting interaction with the Wdr82 component of the HMT complex. These results indicate that the LSD motif mediates the interaction of Setd1b with Rbm15.

**Figure 4:**
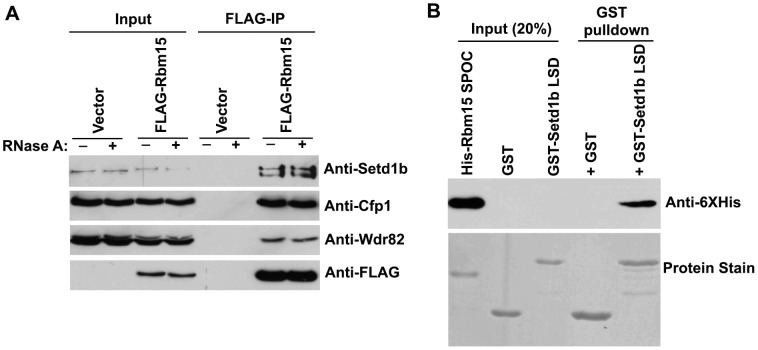
Rbm15 and Setd1b directly interact. A. Inducible T-REx HEK293 cell lines that express FLAG-tagged Rbm15 or carry the empty expression vector were induced with 1 µg/ml doxycycline for three days. Whole cell extracts were prepared and subjected to FLAG immunoprecipitation in the presence (+) or absence (−) of RNase A, and immunoprecipitates were analyzed by Western blotting using the indicated antisera. B. His-SPOC domain (aa 782–977 of Rbm15) and GST or GST-LSD (aa 542–628 of Setd1b) were purified from E. coli cells and analyzed by GST pull-down assay. Proteins were separated by SDS-PAGE and analyzed by Western blotting (top) and Coomassie blue staining (bottom).

The data presented in figures 2 and 3 demonstrate that the Rbm15 SPOC domain and Setd1b LSD domain are necessary for interaction between these proteins. Additional studies were performed to determine whether these domains are sufficient to mediate an interaction. For example, both Rbm15 and Setd1b additionally contain RRM domains, raising the possibility than an RNA moity might also be required for this interaction. However, addition of RNase A to cellular extracts had no effect on the ability of FLAG-Rbm15 to co-immunoprecipitate Setd1b and other HMT components such as Cfp1 and Wdsr82 (Fig. 4A). Furthermore, pull-down studies were performed with isolated Rbm15 SPOC and Setd1b LSD domains purified from E. coli. These experiments demonstrate that GST-Setd1b LSD domain will pull-down His-tagged Rbm15 SPOC domain (Fig. 4B), thus demonstrating that the interaction between Rbm15 and Setd1b is direct.

### The Leukemogenic Rbm15-Mkl1 Fusion Protein Interacts with the Setd1b H3-Lys4 Methyltransferase Complex

**Figure 5:**
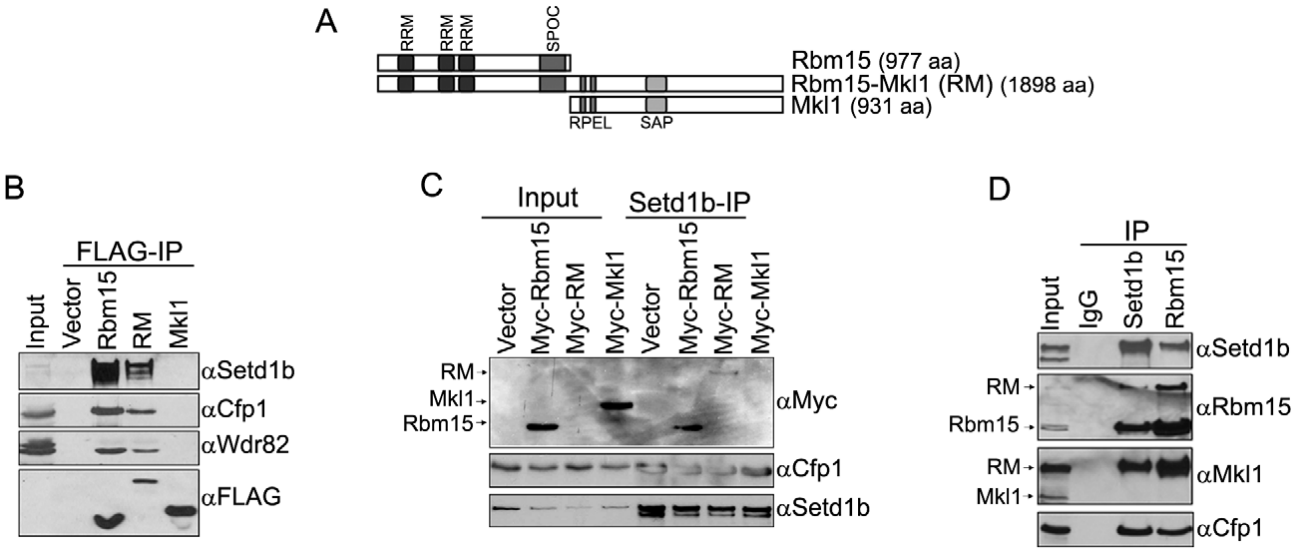
The leukemogenic Rbm15-Mkl1 fusion protein associates with the Setd1b HMT complex. A. Schematic diagram of Rbm15, Rbm15-Mkl1 fusion protein, and Mkl1. Numbers indicate aa residues. B. Nuclear extracts from T-REx HEK293 stable cell lines that express FLAG-tagged Rbm15, Rbm15-Mkl1 (RM), or Mkl1 (or carrying empty vector) were subjected to FLAG immunoprecipitation, and immunoprecipitates were analyzed by Western blotting using the indicated antisera. C. Constructs that express empty vector, Myc-Rbm15, Myc-Rbm15-Mkl1, or Myc-Mkl1 were transiently transfected into HEK293 cells. Nuclear extracts were prepared and subjected to immunoprecipitation using anti-Setd1b antiserum. Immunoprecipitates (Setd1b-IP) were analyzed by Western blotting using the indicated antisera. D. Whole cell extracts from murine 6133 megakaryoblastic leukemic cells were subjected to immunoprecipitation using antisera directed against Setd1b or Rbm15. Immunoprecipitates were analyzed by Western blotting using the indicated antisera.

The leukemogenic Rbm15-Mkl1 fusion protein possesses all of the functional domains of both Rbm15 and Mkl1, including an intact SPOC domain (Fig. 5A). To investigate whether Rbm15-Mkl1 also interacts with the Setd1b HMT complex, constructs that express FLAG-tagged Rbm15, Rbm15-Mkl1, or Mkl1 were transiently expressed in HEK293 cells, and protein interactions were analyzed by co-immunoprecipitation and Western blotting. Figure 5B demonstrates that both FLAG-Rbm15 and FLAG-Rbm15-Mkl1 interact with endogenous Setd1b and associated HMT complex components, but FLAG-Mkl1 alone does not (Fig. 5B). Reciprocal immunoprecipitations were also performed using myc epitope-tagged proteins. Immunoprecipitation using anti-Setd1b antiserum reveals that endogenous Setd1b interacts with myc-Rbm15 and myc-Rbm15-Mkl1, but not with myc-Mkl1 (Fig. 5C).

A stem cell factor (SCF)-dependent murine megakaryoblastic leukemic cell line (6133) was previously derived from a knock-in mouse model of AMKL, in which Rbm15-Mkl1 fusion transcript is expressed from the endogenous murine *RBM15* gene promoter [30]. These cells retain one normal *RBM15* allele. To investigate the interaction between Rbm15-Mkl1 and Setd1b under physiologic conditions, extracts from 6133 cells were immunoprecipitated using antisera directed against Setd1b or Rbm15, and immunoprecipitates were analyzed by Western blotting. As expected, Rbm15 antibody detects both Rbm15 and the larger Rbm15-Mkl1 (RM) fusion protein, and Mkl1 antibody detects both Mkl1 and the Rbm15-Mkl1 fusion protein (Fig. 5D, input lane). Western blotting indicates that Setd1b immunoprecipitates Rbm15 and Rbm15-Mkl1, but not Mkl1. Rbm15 immunoprecipitation shows that Rbm15 interacts with Setd1b and the Setd1 HMT complex component Cfp1, but not with Mkl1. Consistent with the data presented in figure 3B, these results indicate that the Setd1b HMT complex interacts with both Rbm15 and the Rbm15-Mkl1 fusion protein in 6133 leukemic cells.

**Figure 6:**
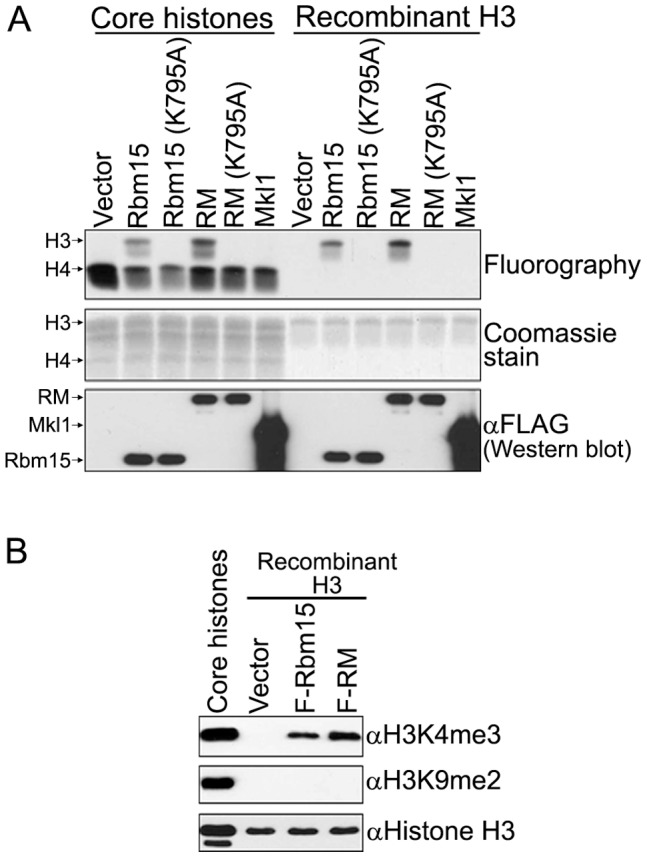
The leukemogenic Rbm15-Mkl1 fusion protein associates with histone H3-Lys4 methyltransferase activity. A. Nuclear extracts isolated from inducible T-REx HEK293 stable cell lines that express FLAG-Rbm15, FLAG-Rbm15 (K795A), FLAG-Rbm15-Mkl1, FLAG-Rbm15-Mkl1 (K795A), FLAG-Mkl1, or carry empty vector were subjected to FLAG immunoprecipitation and bound proteins were eluted by FLAG peptide after extensive washing. HMT activity was assayed by incubating immunoprecipitates with core histones or recombinant histone H3 in the presence of [^3^H]methyl-S-adenosyl methionine. Reaction products were resolved by SDS-PAGE and examined by Coomassie blue staining or fluorography. B. Recombinant human histone H3 purified from *E.coli* was incubated with immunoprecipitates in the presence of methyl-S-adenosyl methionine. Reaction products were analyzed by Western blotting using the indicated modification-specific antibodies. Core histones purified from HEK293 cells were used as a positive control.

**Figure 7:**
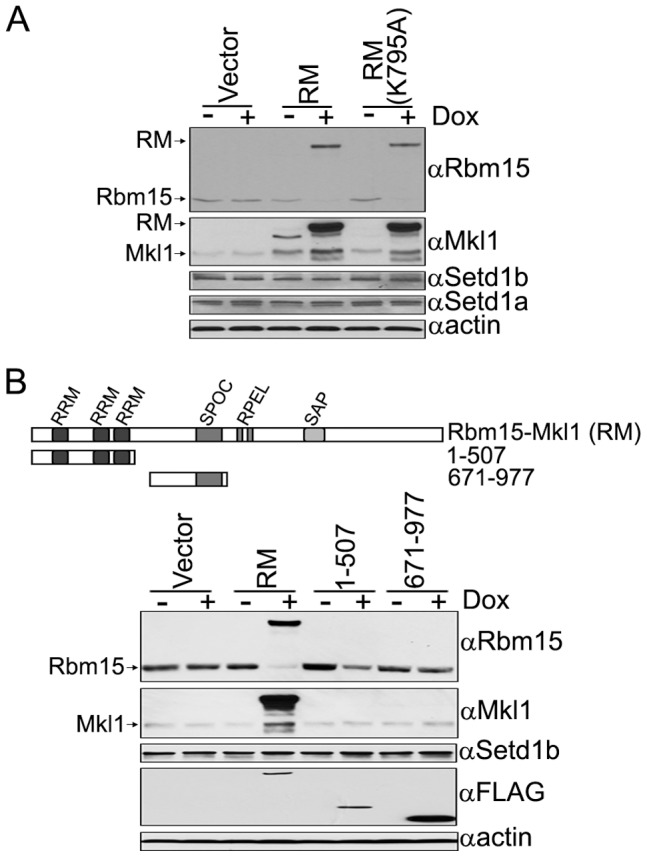
Rbm15-Mkl1 fusion protein decreases the steady-state level of Rbm15. A. Inducible T-REx HEK293 cell lines that express FLAG-tagged Rbm15-Mkl1 (RM), Rbm15-Mkl1 (K795A), or carry the empty expression vector were induced with 0.5 ug/ml doxycycline for three days. The expression level of each protein before and after induction was analyzed by Western blotting using the indicated antisera. Arrows indicate the position of Rbm15, Mkl1, and Rbm15-Mkl1. B. Inducible T-REx HEK293 cell lines that express FLAG-tagged Rbm15-Mkl1, Rbm15 (aa 1–507), Rbm15 (aa 671–977), or carry the empty expression vector were induced with 0.5 ug/ml doxycycline for three days. Whole cell extracts were prepared and analyzed by Western blotting using the indicated antisera. Arrows indicate endogenous Rbm15 and Mkl1.

To investigate whether Rbm15-Mkl1 fusion protein associates with histone H3-Lys methyltransferase activity in a Setd1b-dependent manner, the Setd1b-interaction defective K795A mutation of Rbm15 was introduced into the Rbm15-Mkl1 fusion protein and introduced into inducible HEK293 cells. As expected, the K795A mutation disrupts the interaction of Rbm15-Mkl1 with the endogenous Setd1b HMT complex in HEK293 cells (data not shown). FLAG immunoprecipitates were analyzed for HMT enzymatic activity by incubating with core histones or recombinant histone H3 in the presence of [^3^H]methyl-S-adenosyl methionine. Figure 6A shows that FLAG immunoprecipitates from cells expressing FLAG-Rbm15 or FLAG-Rbm15-Mkl exhibit histone H3 methyltransferase activity, but immunoprecipitates from cells expressing FLAG-Rbm15 (K795A), FLAG-Rbm15-Mkl1 (K795A), or FLAG-Mkl1 do not. The signal for histone H4 methylation, also present in the vector control, is presumably due to non-specific FLAG immunoprecipitation of the PRMT H4 HMT complex, as previously observed [34]. Products of the recombinant histone H3 reactions were further analyzed by Western blotting using histone H3 methylation-specific antibodies, which confirms that the associated HMT activity is histone H3-Lys4 specific (Fig. 6B)**.** These results demonstrate that the leukemogenic Rbm15-Mkl1 fusion protein associates with histone H3-Lys4 methyltransferase activity mediated by the Setd1b complex.

**Figure 8:**
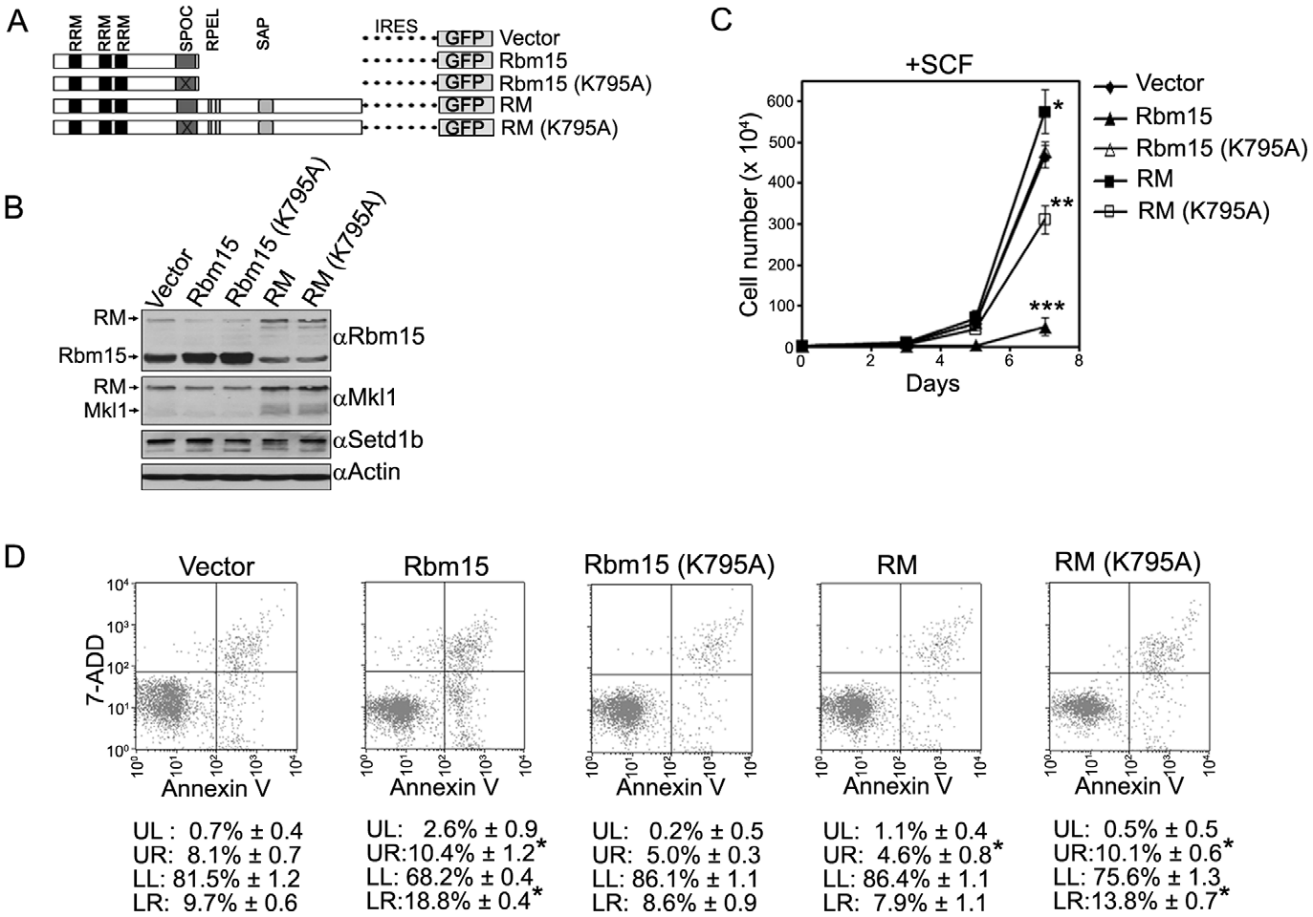
Rbm15-Mkl1 enhances the proliferation of murine 6133 megakaryoblastic leukemic cells in a SPOC domain dependent manner. A. Diagram of pCL6IEGwo lentiviral expression constructs. GFP is expressed from an internal ribosome entry site (IRES). B. Murine 6133 megakaryoblastic leukemic cells were transduced with lentivirus encoding Rbm15, Rbm15 (K795A), Rbm15-Mkl1, Rbm15-Mkl1 (K795A), or with empty vector. After four days of transduction, GFP-positive cells were sorted and cultured in medium containing 10 ng/ml SCF. Whole cell extracts were prepared and analyzed by Western blotting using the indicated antisera. Arrows indicate the position of Rbm15, Mkl1, and Rbm15-Mkl1. C. GFP-positive 6133 megakaryoblastic leukemic cells were cultured in medium containing SCF and cells were enumerated every two or three days after staining with trypan blue. The error bars indicate the mean standard deviation from three experiments, and asterisks indicate *p* values compared to vector control (*, *p* = 0.008; **, *p* = 0.001, ***, *p* = 0.0001). D. Analysis of apoptosis in transduced 6133 megakaryoblastic leukemic cells. GFP-positive 6133 leukemic cells were analyzed for apoptosis using APC-annexin V and 7-ADD staining and flow cytometry. UR, upper right panel corresponding to dead cells; LL, lower left panel corresponding to healthy cells; LR, lower right panel corresponding to apoptotic cells. Numeric values represent a summary of data from three experiments. Asterisks indicate *p* values compared to vector control (*p*<0.05).

### Expression of the Rbm15-Mkl1 Fusion Protein Decreases the Steady-state Level of Endogenous Rbm15 and Increases Mkl1 Expression

Further analysis of HEK293 stable cell lines that express Rbm15-Mkl1 or Setd1b-interaction defective Rbm15-Mkl1 (K795A) reveals that endogenous Rbm15 expression is decreased ∼8-fold, while Mkl1 expression is increased ∼3-fold, upon induction of either of these fusion proteins (Fig. 7A). However, the expression levels of Setd1b and Setd1a are not changed. Additional studies revealed that expression of Rbm15 does not affect Mkl1 expression, and expression of Mkl1 does not affect Rbm15 expression (data not shown). Figure 7B demonstrates that expression of the N-terminal (1–507 aa) region of Rbm15, encompassing three RRM domains, is sufficient to decrease endogenous Rbm15 expression without changing Mkl1 expression. Expression of the C-terminal (671–977 aa) region of Rbm15, which contains the SPOC domain, does not change expression of endogenous Rbm15 or Mkl1 proteins. These results indicate that the N-terminal domain of Rbm15 regulates the steady-state level of endogenous Rbm15 by currently unknown mechanisms, a phenomenon that is independent of interaction with the Setd1b HMT complex.

### Rbm15-Mkl1 Regulates Cellular Proliferation and Survival of Murine 6133 Megakaryoblastic Leukemic Cells in a SPOC Domain-dependent Manner

SCF-dependent murine 6133 megakaryoblastic leukemic cells were transduced with lentivirus encoding Rbm15, Rbm15 (K795A), Rbm15-Mkl1, Rbm15-Mkl1 (K795A), or the empty vector. Each vector also encodes an enhanced green fluorescent protein (GFP) tracer (Fig. 8A). GFP-positive cells were isolated and protein expression was analyzed by Western blotting. Figure 8B demonstrates that forced expression of Rbm15 or Rbm15 (K795A) in 6133 cells causes a 2.5-fold decrease of Rbm15-Mkl1 fusion protein, and forced expression of Rbm15-Mkl1 or Rbm15-Mkl1 (K795A) leads to a 2.3- fold decrease of Rbm15 and a 3.6-fold increase of Mkl1. Consistent with observations in inducible T-REx HEK293 cells (Fig. 7), these results indicate that the expression level of Rbm15 and Rbm15-Mkl1 is reciprocally regulated, and expression of the Rbm15-Mkl1 fusion protein increases Mkl1 expression.

**Figure 9:**
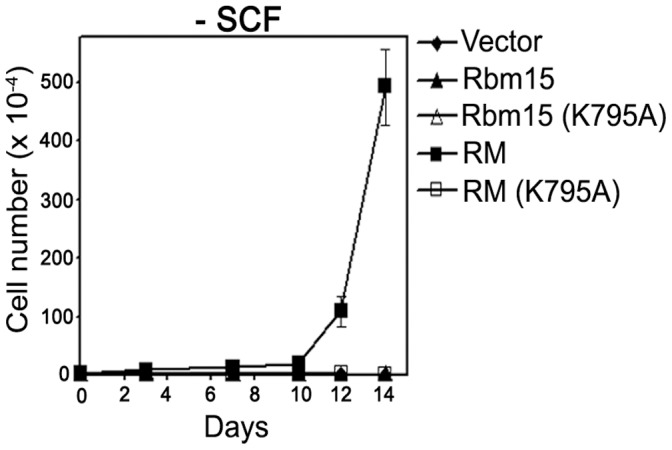
Over-expression of Rbm15-Mkl1 transforms murine 6133 megakaryoblastic leukemic cells in a SPOC domain dependent manner. Following sorting, GFP-positive cells were cultured in the presence of SCF for several days. Cells were then plated in medium lacking SCF, and cells were enumerated every two or three days after staining with trypan blue. The error bars indicate the mean standard deviation from three experiments.

Further studies were performed to assess the functional significance of Rbm15-Mkl1 in cellular proliferation and survival. 6133 cells were transduced with lentiviral vectors as described above, and GFP-positive cells were cultured in the presence of SCF and viable cells were counted (Fig. 8C). Cells transduced with Rbm15-Mkl1 exhibited enhanced proliferation. However, Rbm15-transduced cells that express reduced levels of Rbm15-Mkl1 showed markedly decreased growth, suggesting that Rbm15-Mkl1 stimulates proliferation of 6133 leukemic cells. Expression of the Setd1b-interaction defective form of Rbm15-Mkl1 (K795A) inhibited growth of 6133 leukemic cells, while Setd1b-interaction defective Rbm15 failed to inhibit growth of 6133 leukemic cells. These results indicate that the presence of an intact SPOC domain is required for the stimulation of proliferation mediated by Rbm15-Mkl1, possibly indicating a requirement for interaction between Rbm15-Mkl1 and Setd1b.

To further investigate the growth stimulatory effects of the Rbm15-Mkl1 fusion protein, we analyzed levels of apoptosis for GFP-positive cells. 6133 cells transduced with Rbm15-Mkl1 showed a significant decrease of dead (4.6% vs 8.1%, *p* = 0.0006) and apoptotic (7.9% vs 9.7%, *p* = 0.014) cells compared to control cells. However, 6133 cells transduced with either Rbm15 or the SPOC domain mutated form of Rbm15-Mkl1 (K795A) exhibit significantly elevated levels of dead (10.4% vs 8.1%, *p* = 0.01; 10.1% vs 8.1%, *p* = 0.02, respectively) and apoptotic (18.8% vs 9.7%, *p* = 0.001; 13.8% vs 9.7%, *p* = 0.008, respectively) cells compared to control cells (Fig. 8D). These results indicate that Rbm15-Mkl1 enhances cell proliferation by supporting cell survival, an activity that requires an intact SPOC domain that interacts with the Setd1b HMT complex.

### Over-expression of Rbm15-Mkl1 Renders Murine 6133 Megakaryoblastic Leukemic Cells Factor-independent in a SPOC Domain Dependent Manner

Previous studies demonstrated that SCF-dependent 6133 leukemic cells are further transformed by expression of the thrombopoietin receptor mutant Mpl^W515L^, resulting in cytokine-independent growth [30]. To investigate whether elevated expression of the Rbm15-Mkl1 fusion protein is sufficient to confer factor-independent growth to 6133 leukemic cells, 6133 cells were transduced with lentiviral vectors as described above, and GFP-positive cells were cultured in the absence of SCF (Fig. 9). Surprisingly, Rbm15-Mkl1-transduced cells grew in the absence of SCF. However, other transduced cells, including those expressing SPOC domain-defective Rbm15-Mkl1 (K795A), did not. These results indicate that enhanced expression of the Rbm15-Mkl1 fusion protein is sufficient for cytokine-independent proliferation of 6133 leukemic cells, an effect that is mediated by the SPOC domain of Rbm15-Mkl1.

## Discussion

A large number of enzymes have been identified that add or remove epigenetic marks on chromatin. However, the mechanisms that control the genomic targeting of these epigenetic modifying enzymes are not well understood. The Setd1a and Setd1b HMT complexes are closely related yet exhibit distinct sub-nuclear distributions, thus providing an informative system with which to study this important question. Co-immunoprecipitation studies were performed to identify molecules that interact specifically with Setd1a or Setd1b, thus revealing putative targeting molecule candidates. We report here that Setd1b interacts specifically with both Rbm15 and the leukemogenic Rbm15-Mkl1 fusion protein that is frequently found in non-Down syndrome pediatric AMKL.

Previous work established that expression in mice of a knock-in allele encoding the Rbm15-Mkl1 fusion protein leads to AMKL at a low penetrance and long latency period, suggesting that Rbm15-Mkl1 expression is not sufficient for leukemogenesis and that cooperating mutations are needed for the development of AMKL [30]. Consistent with this hypothesis, introduction of a constitutively active form of the Mpl receptor into bone marrow cells cooperates with Rbm15-Mkl1 and leads to myeloproliferative disease with a high frequency. Similarly, introduction of the activated form of the Mpl receptor into 6133 cells expressing Rbm15-Mkl1 leads to further transformation and SCF-independent growth, which is associated with activation of the Mpl/MAPK signaling pathway [30]. Remarkably, we find that forced expression of the Rbm15-Mkl1 fusion protein is sufficient to drive SCF-independent growth of 6133 cells. This effect is dependent on the presence of an intact SPOC domain of Rbm15-Mkl1, which we demonstrate interacts with the Setd1b HMT complex. Further work will be required to determine what biochemical perturbations transform 6133 cells as a consequence of forced expression of Rbm15-Mkl1.

How the Rbm15-Mkl1 fusion protein specifically induces megakaryoblastic leukemia is not clear. We report here that over-expression of the Rbm15-Mkl1 fusion protein leads to decreased Rbm15 expression and increased Mkl1 expression in both non-hematopoietic and megakaryoblastic leukemic cells. Importantly, previous genetic knockout and siRNA-mediated depletion studies revealed that Rbm15 inhibits myeloid and megakaryocytic differentiation, suggesting that dysregulation of Rbm15-dependent pathways such as Notch signaling may contribute to the development of AMKL [13]–[15]. Furthermore, Mkl1 expression increases during megakaryocytic differentiation, and genetic knockout and forced expression of Mkl1 in hematopoietic cells reveal that Mkl1 promotes megakaryocytic differentiation [40]. Thus, decreased Rbm15 expression and increased Mkl1 expression following Rbm15-Mkl1 fusion protein expression may promote megakaryocytic development and eventually contribute to the development of AMKL. It is important to note, however, that repression of Rbm15 is not sufficient to induce factor-independent growth of 6133 cells, because the SPOC domain-mutated form of Rbm15-Mkl1 (K795A) represses Rbm15 expression but fails to support 6133 cell proliferation in the absence of SCF. Whether modulation of Rbm15 and Mkl1 expression are required for the development of AMKL, and elucidation of the molecular mechanisms that regulate Rbm15 and Mkl1 expression will require additional studies.

The Rbm15 SPOC domain has been reported to interact with several factors, including histone deacetylase 3 and the mRNA export factors Nxf1 and Dbp5 [11], [12], [41], [42], suggesting that the Rbm15 SPOC domain mediates molecular interactions involved in transcriptional activation, transcriptional repression, and RNA metabolism. How the SPOC domain specifically interacts with appropriate effector molecules *in vivo* is not understood. We have not determined whether the K795A SPOC domain mutation within the Rbm15-Mkl1 fusion protein affects other interactions in addition to Setd1b. Therefore, we cannot rule out the possibility that additional Rbm15-Mkl1 SPOC domain interactions might be relevant to the observed transforming activity. However, dysregulation of the Mll1 member of the Set1 HMT family is commonly associated with myeloid leukemia. Thus, the findings reported here suggest a model in which the leukemogenic activity of the Rbm15-Mkl1 fusion protein may be a consequence of altered regulation and/or genomic targeting of Setd1b, resulting in altered epigenetic regulation of critical regulatory genes controlling megakaryocytic development. Thus, identification of small molecule inhibitors of the interaction between Rbm15-Mkl1 and Setd1b might provide novel therapeutic strategies for the treatment of AMKL.
